# Lightweight three-factor authentication protocol for 6G-enabled healthcare systems using Chebyshev chaotic maps and BioHashing

**DOI:** 10.1038/s41598-025-31701-y

**Published:** 2026-01-08

**Authors:** Amr Magdy Abbas, Mohamed E. Nasr, Roayat Ismail Abdelfatah

**Affiliations:** 1 Electronics and Electrical Communications Engineering Department, Faculty of Engineering, Tanta University, Tanta, 31527 Egypt; 2 Electrical Engineering Department, College of Engineering, Prince Sattam Bin Abdulaziz University, Al-Kharj, 16278 Saudi Arabia

**Keywords:** Computational biology and bioinformatics, Engineering, Health care, Mathematics and computing

## Abstract

In the evolving field of intelligent healthcare systems, secure and efficient communication is critical. This paper proposes a three-factor authentication protocol designed for 6G-enabled healthcare environments that merges smart cards, passwords, and biometrics to ensure robust security. To enhance performance, the protocol makes use of Chebyshev chaotic maps for key generation and lightweight encryption techniques. It introduces a fast authentication mechanism through parameter reuse achieving reduced latency and eliminates the need for a pre-established secure channel during registration, making deployment more practical. Furthermore, the scheme incorporates a certificate-less, user-centric framework for multi-server healthcare systems. Formal security verification using Scyther confirms the protocol’s resilience to various attacks, including replay, man-in-the-middle, and impersonation attacks. Comparative analysis demonstrates its effectiveness in terms of security, communication efficiency, and computational cost, achieving an average authentication time of **0.00354 ms** and a 57% reduction in storage cost. These results make it a viable solution for healthcare applications in resource-constrained and latency-sensitive environments.

## Introduction

In recent years, there has been a growing focus on enhancing security within healthcare systems. The transmission of patient data over open network introduces vulnerabilities, as open network is susceptible to various attacks, potentially compromising patient privacy^1–4^ Consequently, it is imperative to ensure that patient information is transmitted over secure channels to safeguard against unauthorized access. Ensuring robust security mechanisms in healthcare systems is essential to mitigate potential risks. Traditional password-based and two-factor authentication methods, which combine passwords with smart cards, have been widely adopted to address security concerns. However, these approaches exhibit notable vulnerabilities. For instance, if an adversary obtains a user’s password, they can exploit a stolen smart card to execute attacks, undermining the system’s security^5–7^. The increasing demand for high data rates and low latency in healthcare applications has highlighted the limitations of existing communication technologies such as 3G, 4G and even 5G. These technologies may not adequately support advanced applications such as holographic communications and other data-intensive services that are essential to modern healthcare systems. To address these challenges, the development of sixth-generation (6G) wireless communication systems is underway and promises to meet the stringent requirements of future healthcare applications by offering increased data rates, reduced latency, and improved security features^8–10^. The integration of 6G technology into healthcare systems is expected to revolutionize medical services by providing robust support for applications that demand high data throughput and real-time responsiveness. This advancement will facilitate the secure and efficient exchange of large volumes of patient data, thereby improving diagnostic accuracy, enabling advanced telemedicine services, and ensuring that patient privacy is maintained across all communication channels^11,12^.

To address the limitations of two-factor authentication mechanisms, researchers have introduced Three-factor authentication protocols for Internet-enabled wireless sensor networks. These protocols combine smart cards, passwords, and biometric data to strengthen security. For example, an authenticated key agreement protocol for 5G-based telemedicine systems^1^ has been proposed. Additionally, a three-factor authentication and key agreement protocol for multiple service providers in 6G-enabled intelligent healthcare. systems has been suggested, incorporating a time-bound property to ensure fast and secure communication^9^ Another approach employs elliptic curve cryptography (ECC) to integrate biometrics, smart cards, and passwords, using a challenge-response mechanism to further enhance security^13^. Furthermore, a lightweight and anonymous three-factor authentication protocol for wireless sensor networks has been introduced, aimed at providing robust security against various threats while maintaining efficiency^14^. Other studies have proposed a Three-Factor Fast Authentication Scheme with Time Bound and User Anonymity for Multi-Server E-Health Systems in 5G-based Wireless Sensor Networks^15^.

### Our contribution and paper organization

This paper presents a protocol designed to maintain user identity confidentiality while enabling robust mutual authentication between patients and healthcare providers over a public channel. Utilizing three-factor authentication, the protocol ensures the secure establishment of a reliable shared session key. Additionally, we incorporate a lightweight encryption technique to enhance the protocol’s suitability for 6G applications. The significant findings of this study are summarized below.

- Our protocol is designed to work effectively with 6G networks, taking advantage of their advanced features^16^. While 5G was already useful for smart healthcare applications, 6G provides greater opportunities for developing intelligent healthcare systems. This new technology enables real-time, AI-powered medical services across various providers, improving patient care with smart, interconnected devices and high-speed connectivity. For example, 6G can support remote surgeries where surgeons operate on patients from different locations with minimal delay. Additionally, AI can enhance emergency responses by quickly analyzing patient data to prioritize urgent cases.
- In our scenario involving a patient and a healthcare system with multiple services provided by various servers^17^, the patient must follow some steps to join our system. First is to obtain a smart card from our system. After receiving the card, they begin to compute a shared secret using the Chebyshev operation. which is known for its robustness in secure communications. This shared key is then used to begin the registration process through a public channel, eliminating the need for a secure channel during registration and making the process more efficient and accessible.
- Since our protocol relies on three-factor authentication, it ensures a high level of security by combining three essential elements: a password (something they know), biometric data (something they are), and the smart card (something they have) which reduce the risk of unauthorized access. After registering with these three factors, the server sends a signed message that the patient will use to log in. Our authentication process uses lightweight operations, making it suitable for low power 6G devices.
- Additionally, our protocol introduces a fast authentication technique that allows for the computation of a new shared secret with fewer operations and previous parameters from the last session. We also enable the user to update their password for enhanced security. Since the user’s credentials are stored on the smart card, they only need to sign in once, even if they want to access multiple services.
- Finally, we utilized Wolfram Mathematica to validate the protocol and confirmed its security proof using the Scyther tool. Additionally, we conducted comparisons with other research studies in terms of various factors such as communication cost, storage size, and number of operations. These comparisons ensure that our protocol is robust and performs effectively in comparison to existing solutions.

The proposed scheme advances the state of the art in three-factor authentication by introducing a lightweight and secure protocol specifically tailored for 6G-enabled healthcare systems. Unlike conventional approaches, our method integrates smart cards, biometric credentials, and passwords with a novel Chebyshev chaotic map–based key generation mechanism to enhance computational efficiency and randomness. In addition, we incorporate a security-enhanced BioHashing technique for biometric template protection, which mitigates risks of biometric compromise and replay attacks. Furthermore, the protocol leverages session parameter reuse and optimized message flows, effectively reducing computational cost and communication overhead without sacrificing security.

To contextualize these contributions, Table 1 presents a comparative summary of recent state-of-the-art three-factor authentication schemes alongside our proposed work. The comparison highlights the algorithms employed, their achievements, and their limitations, clearly demonstrating how our scheme addresses existing gaps while achieving real-time efficiency and robustness.

**Table 1:** State-of-the-art comparison in three-factor authentication schemes.

Author (year)	Algorithms used	Achievements	Limitations
Le et al. (2022)^9^	Rabin cryptosystem (modular squaring), one-way hash, Biohash function, time-bound property.	Strong resistance to replay and impersonation attacks; suitable for IoT devices	Registration via secure channel; relies on smart card; signatures/non-repudiation not achieved
Huang (2024)^13^	ECC, Fuzzy Extractor	Robust WSN authentication; biometric integration.	Slow (6.6 ms); no 6G optimization.
Wong et al. (2020)^15^	BioHashing with SHA-256, smart card	Enhanced biometric template protection; improved authentication accuracy	High storage requirement for biometric templates; not optimized for constrained devices
Lin et al. (2021)^18^	ECC, Smartcard	Privacy-preserving telemedicine.	Secure channel needed; high storage (2,176 bits).
Our Work (2024)	Chebyshev maps, BioHash, XOR	6G-optimized; fastest authentication; no secure channel; Scyther-verified.	Dependent on accurate clock synchronization; relies on tamper-resistant smart cards

The remainder of the paper is organized as follows: preliminary is given in Section "Preliminary", the proposed scheme construction is given in Section "Proposed system construction"; security analysis is given in Section "Security analysis"; formal security evaluation using Scyther is given in Section "Comprehensive security validation", performance analysis is given in Section "Performance analysis" and finally, conclusion is given in Section "Conclusion".

## Preliminary

In this section, we will start by outlining the cryptographic techniques that are key to our protocol, which aims to establish a secure shared key and ensure mutual authentication between patient and healthcare systems. Then, we will show all the notations utilized throughout the research. Then analyzing vulnerabilities in prior work. Finally, we will present our proposed protocol, which is structured into several phases, designed to achieve secure communication and reliable authentication.

### Cryptographic tools

In this section, we outline the cryptographic tools utilized in our protocol to ensure both security and efficiency. We employ BioHash and the **Chebyshev polynomial** for generating the shared key, which allows communication to be done over public channel and digital signatures, providing a robust layer of security. Additionally, our protocol incorporates **rotation**, **XOR**, and **modular addition** operations. These techniques are considered lightweight methods, making our protocol not only secure but also fast, particularly suited for applications requiring high-speed processing.

#### BioHash function

BioHashing is employed to transform a biometric template (BIO) into a fixed-length binary string securely bound to a user-specific secret key K. In our protocol, the BioHash function is defined as:

$$\:{h}_{bio}(BIO,K)=Trun{c}_{256}\left(H\right(K \oplus BIO\left)\right)$$

where H: {0,1}* → {0,1}⁵¹² is a cryptographic hash function (e.g., SHA-512), Trunc₂₅₆ selects the first 256 bits of the hash output, and ⨁ denotes bitwise XOR.

This formulation represents a lightweight instantiation of the traditional BioHashing scheme, in which the random projection stage is substituted with a keyed hash operation to reduce computational complexity and latency.

Although BioHashing is sometimes described as noise-tolerant, it does not inherently include any error-correction mechanism. When the biometric input varies significantly (e.g., due to sensor noise or environmental changes), the resulting hash may differ completely because of the avalanche property of the cryptographic hash function. Consequently, the BioHash process by itself cannot guarantee successful recognition under high-noise conditions.

In our implementation, robustness is achieved not within the BioHash function itself but through the feature extraction and normalization stages. These preprocessing steps ensure that noisy biometric data are converted into stable and consistent templates before hashing. Furthermore, modern biometric sensors integrated into intelligent healthcare devices (such as fingerprint or iris modules with liveness detection) produce reliable and high-quality BIO readings during both enrollment and authentication phases. As a result, the input to $\:{h}_{bio}(.)$remains sufficiently consistent to ensure stable verification outcomes.

Unlike fuzzy extractors, which rely on helper data and error-correcting codes to reconstruct consistent keys from noisy inputs, BioHashing provides a computationally lightweight and fast alternative. This makes it particularly suitable for resource-constrained 6G-enabled healthcare systems, where low latency and energy efficiency are critical design objectives^19–21^.

#### Chebyshev chaotic map

In our protocol, we use Chebyshev chaotic map for generating the shared key, which allows communication to be done over public channel in registration phase as well as digital signature.

Consider a large prime number p, a real number ϕ within the interval [-1,1], an integer ω, and The Chebyshev output denoted by y. The Chebyshev Polynomial of degree ω. represented as $\:{T}_{\omega\:}\left(\phi\:\right)$, is defined according to Eq. (1). The polynomial follows a specific recurrence relation, detailed in Eq. (2):

1 $$\:{T}_{\omega\:}\left(\alpha\:\right)=\text{c}\text{o}\text{s}(\omega\:.{\text{c}\text{o}\text{s}}^{-1}\left(\alpha\:\right))\:$$

2 $$\:{T}_{\omega\:}\left(\alpha\:\right)=\left\{\begin{array}{c}1\:\:\:\:\:\:\:\:\:\:\:\:\:\:\:\:\:\:\:\:\:\:\:\:\:\:\:\:\:\:\:\:\:\:\:\:\:\:\:\:\:\:\:\:\:\:\:\:if\:\omega\:=0\\\:\alpha\:\:\:\:\:\:\:\:\:\:\:\:\:\:\:\:\:\:\:\:\:\:\:\:\:\:\:\:\:\:\:\:\:\:\:\:\:\:\:\:\:\:\:\:\:\:\:\:\:if\:\omega\:=1\\\:2\alpha\:.{T}_{\omega\:-1}\left(\alpha\:\right)-{T}_{\omega\:-2}\left(\alpha\:\right)\:\:\:\:\:\:if\:\omega\:\ge\:1\end{array}\right.\:$$

To enhance the properties of the Chebyshev chaotic map, a modified Chebyshev polynomial is utilized, as defined in Eq. (3). Here, g is assumed to be a generator of the prime p, a detailed explanation of this map can be found in^19,20^.

3 $$\:y={T}_{g}\left(\alpha\:\right)\:mod\:p$$

The Chebyshev chaotic map performs exceptionally well when paired with other cryptographic functions, owing to its strong randomness and intricate dynamic properties. This effectiveness is demonstrated in the following cryptographic challenges:

##### Discrete logarithm problem (DLP)

Given two elements y and $\:\phi\:$, it is extremely difficult to compute the value of g that satisfies Eq. (3).

##### Computational Diffie-Hellman problem (CDHP)

Due to the chaotic map’s properties, it is computationally impractical to solve Eq. (4) to determine the value of $\:{T}_{g\omega\:}\left(\alpha\:\right)$ for any value of, $\:{T}_{\omega\:}\left(\alpha\:\right)\:$, $\:{T}_{g}\left(\alpha\:\right)$.

4 $$\:{T}_{\omega\:}\left({T}_{g}\left(\alpha\:\right)\right)\equiv\:{T}_{g}\left({T}_{\omega\:}\left(\alpha\:\right)\right)\equiv\:{T}_{g\omega\:}\left(\alpha\:\right)\:mod\:p$$

#### Rotation using RR method

Comprehensive details on this rotation technique are provided in^22,23^. Using the RR method, the parameters (A) and (B) are rotated through the following steps:

- Calculating the length of the B parameter: L, then find the modular arithmetic of the B parameter with respect to L (B mod L).
- execute the XOR operation between the parameters A and B (A⊕B).
- Applying a left circular shift to (A⊕B) (B mod L) The outcome of this step is defined as Rot (A, B).

Additionally, the function RRot (A, B) operates as the inverse of Rot (A, B).

#### Local nonce generation

In the proposed scheme, we employ a *local nonce* mechanism to ensure message freshness without increasing the transmitted data size. Unlike traditional nonce exchange methods, where a random value is explicitly sent between entities, the local nonce is **independently derived** at both the device and the server using a shared secret key and session-specific parameters.

The local nonce $\:{n}_{i}$is computed as:

$$\:{n}_{i}={\text{T}\text{r}\text{u}\text{n}\text{c}}_{64}\left({\text{H}\text{M}\text{A}\text{C}}_{{K}_{s}}\right(I{D}_{i}\parallel\:{t}_{i}\parallel\:ct{r}_{i}\left)\right)$$

where $\:{K}_{s}$is the shared secret key, $\:I{D}_{i}$is the device identity, $\:{t}_{i}$is the current timestamp, and $\:ct{r}_{i}$is a local counter that increments with each authentication attempt. This implicit derivation guarantees that both sides generate the same nonce value without any additional transmission overhead.

The inclusion of the local nonce increases robustness against replay and timing-based DoS attacks while preserving the lightweight nature of the protocol. Since the nonce is never transmitted, it does not affect bandwidth consumption or message structure.

### Notations

Table 2 provides definitions for all the parameters discussed in this paper, including their symbols as used in the cryptographic functions. This table is intended to facilitate a better understanding of the proposed protocol.

**Table 2:** Notation and definitions used in the proposed protocol.

Symbols	Descriptions
$\:{s}_{j}$	$\:{\varvec{j}}^{\varvec{t}\varvec{h}}$ *server*
$\:{p}_{i}$	$\:{\varvec{i}}^{\varvec{t}\varvec{h}}$ patient
$\:{ID}_{i}$	Identity of patient
$\:{PW}_{i}$	Password of patient
$\:{B}_{i}$	Biometric of patient
$\:{t}_{i}$	Timestamp
$\:{n}_{i}$	Local nonce
A, C	Public key for patient and server
a, c	Private key for patient and server
S	Shared key between patient and server
$\:\oplus\:$	XOR function
$\:{h}_{bio}(.)$	Biohash function
Mod	Modular function
Rot (,)	Rotation function
$\:{[.]}_{sci}$	Store in smart card
$\:{\left[.\right]}_{DBj}$	Store in server database
$\:{[.]}_{HDi}$	Store in intelligent healthcare device
SK	The first shared session key
$\:{SK}_{NEW}$	The new shared session key
$\:{\text{T}}_{c}\left(w\right)$	Chebyshev function

### Security limitations of existing protocols

To highlight the necessity of our proposed protocol, we carefully reviewed three representative three-factor authentication (3FA) schemes in the literature, namely Huang et al.^13^, Le et al.^9^, Lin et al.^18^, and Wong et al.^15^. Each scheme has made important contributions to the field of secure healthcare communication; however, they still reveal important shortcomings when evaluated under realistic attack scenarios.

- Huang et al.^13^. This protocol is vulnerable to **stolen smart card** attacks, since static credentials stored on the card allow adversaries to perform offline guessing of passwords. Moreover, impersonation becomes possible once the card is compromised. The scheme also faces **scalability issues** in wireless sensor networks, making it impractical in large-scale healthcare environments.
- Le et al.^9^. The protocol lacks an efficient **password update mechanism**. Some static values remain constant across sessions, which introduces the risk of **traceability**; an adversary observing multiple sessions can link them to the same user. This violates anonymity and privacy requirements in 6G-enabled healthcare systems.
- Wong et al.^15^. The scheme does not provide **forward secrecy**, which means that if a long-term secret is compromised, past session keys can be reconstructed. Furthermore, synchronization issues may cause **replay vulnerabilities**, and the protocol remains susceptible to **stolen verifier attacks** where a compromised server database can expose sensitive parameters.
- Lin et al.^18^. This protocol relies heavily on **static credentials** stored on smart cards without strong biometric binding. If a smart card is stolen, adversaries can launch credential-based impersonation attacks, severely undermining patient identity protection.

These weaknesses are summarized in Table 3, alongside the countermeasures applied in our proposed scheme.

**Table 3:** Security limitations of existing protocols and our solutions.

Protocol	Limitations	Our solution
Huang et al.^13^	Vulnerable to stolen smart card, impersonation, and offline guessing; scalability issues in WSNs.	BioHashing protects biometrics; Chebyshev maps strengthen keys; lightweight design fits healthcare devices.
Le et al.^9^	No efficient password update; some static values → risk of traceability.	Secure password update: BioHash + session randomness ensures unlinkability; dynamic parameters resist tracing.
Wong et al.^15^	Lacks forward secrecy; replay risk with desynchronization; vulnerable to stolen verifier.	Scyther tool-ensures forward secrecy; timestamps/nonces block replay; chaotic maps mitigate verifier compromise.
Lin et al.^18^	Stolen smart card attacks: Static credentials stored without biometric binding.	Binds credentials to BioHash and dynamic parameters (Eqs. 11–12).

This comparative analysis demonstrates that existing solutions fail to simultaneously guarantee privacy, unlinkability, and forward secrecy under realistic attack scenarios. In contrast, our proposed protocol incorporates BioHashing and Chebyshev chaotic maps within a lightweight three-factor framework, ensuring resistance against smart card loss, impersonation, replay, and verifier compromise.

## Proposed system construction

This section outlines the system model of the proposed protocol, focusing on the communication capabilities facilitated by a 6G-enabled intelligent healthcare system.

**Fig. 1:**
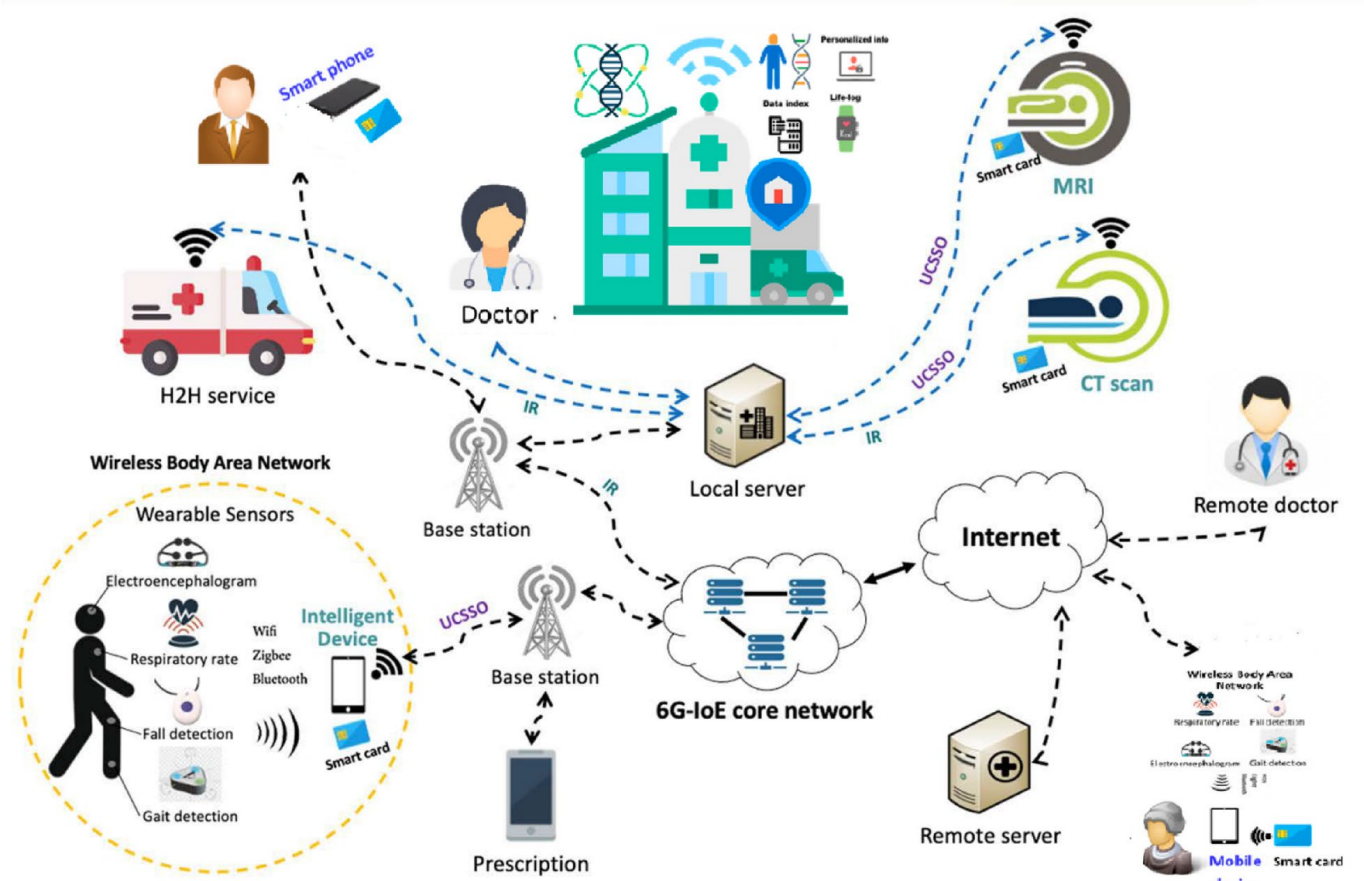
The proposed system model.

### System architecture model

The proposed model comprises two primary entities: the patient (Pi) and the healthcare provider (Sj), communicating within a multi-server environment, as illustrated in (Fig. 1).

The patient (Pi) can perform a Certificate-Less User-Centric Single Sign-On (CL-UCSSO) process, utilizing a single set of credentials saved on their smart card to access services from multiple healthcare providers (Sj) without the need for a central authority. These services encompass a wide range of medical needs, including treatment, prescriptions, doctor-to-doctor consultations (H2H), MRI, CT scans, and more. These services can be offered by In-hospital doctors or distant specialists. For instance, consider the patient monitoring service conducted via a Wireless Body Sensor Network (WBSN). This network includes various medical sensors, such as those measuring respiratory rate or detecting falls, which are worn by the patient (Pi)^9^. In-hospital doctors or distant specialists. The sensors capture essential health data and relay it to the patient’s (Pi) mobile device using wireless technologies such as Wi-Fi. This data is then securely sent to the healthcare provider (Sj) via the 6G network’s IR signal, facilitating further communication and processing. The service enables continuous real-time monitoring of patients, ensuring that patients can go about their normal daily activities without any interruptions or restrictions. With ultra-high data rates, 6G enables faster transmission speeds, particularly for large image files from MRI or CT scanners, facilitating timely treatments. Additionally, Healthcare data may be retained for future use in services like pathology or data analysis. It’s worth mentioning that P_i_’s mobile devices, as well as MRI and CT machines designed for hospital applications, operate as AI-powered intelligent systems. Beyond the smart capabilities of conventional devices, they can autonomously initiate healthcare services upon detecting abnormal symptoms in **Pi**. For instance, if a fall or respiratory depression is detected, the mobile device will trigger an H2H service, whereas the MRI machine will automatically notify the most suitable doctor upon identifying abnormal signal intensity in the images. To guarantee secure communication between Pi and Sj the proposed protocol establishes an authenticated session key, which encrypts the exchanged messages. For this, Pi selects a device for registration with Sj using a combination of Password, biometrics, and smart card, ensuring a robust A three-factor authentication scheme. Additionally, the protocol incorporates a fast Key agreement procedure. Following the initial authentication phase, enabling Pi and Sj to efficiently compute multiple fresh shared keys. This approach Notably enhances the exchange process. While reducing the computational burden on low-power devices in the 6G-enabled Internet of Everything (IoE) healthcare environment.

### Our proposed protocol

In our protocol, the patients use a smart card and a smart device, and the healthcare server securely verifies each other over a public channel. This allows them to create a shared session key, which helps keep their healthcare communication safe and private. This key ensures that all their future exchanges are protected from any potential threats. Following the initial setup phase, rapid authentications are carried out, allowing Pi and Sj to quickly generate new session keys.

additionally, Additionally, Pi can change their password to make it more secure, the protocol is divided into four main phases: The network deployment phase, set up phase, registration, login and authentication, rapid authentication and password modification. Table 2 outlines the notations and cryptographic functions utilized in the protocol.

#### The network deployment phase

In this phase, the healthcare system initiates deployment by generating its public parameters. This process includes selecting a random integer w and a large prime number p. Next, the system determines a secret value c to act as its private key. Leveraging the Chebyshev chaotic map, it computes the corresponding public key C, as expressed in Eq. (5):

5 $$\:\text{C}={\text{T}}_{c}\left(w\right)\:\text{m}\text{o}\text{d}\:\text{p}$$

The parameters used in the key generation (w, p), along with the public key, are then stored on the smart card, which the patient needs to join the system.

#### Set up phase

In this phase, the patient generates a public-private key pair using the discrete Chebyshev map. After selecting a private key, the patient computes the public key according to Eq. (6) with the help of the public parameters stored in the smart device (w, P).

6 $$\:A={\text{T}}_{a}\left(w\right)\:\text{m}\text{o}\text{d}\:$$

Next, the patient computes a shared key with the healthcare system to secure the registration process. This is done over a public channel, utilizing the public key of healthcare stored on the smart device to derive the shared key, as shown in Eq. (7).

7 $$\:S1={\text{T}}_{a}\left(C\right)\:\text{m}\text{o}\text{d}\:\text{p}$$

We extract 256 bits, denoted as S, to be used as the shared key. This size is specifically chosen to ensure optimal compatibility with 6G communication systems, providing a balance between security and performance. A 256-bit key delivers robust cryptographic strength which ensures that the protocol maintains both high security and efficiency, crucial for the low-latency demands of 6G networks.

#### Registration phase

**Before registration**,** the patient follows several steps to authenticate the healthcare system**:

1. First, the patient visits the healthcare website, downloads the published public key, and compares it with the one stored on their smart device to ensure they are not interacting with a fake healthcare provider.
2. Once the values match, the patient generates a challenge as described in Eq. (8), which includes a selected nonce and a timestamp. This ensures that the healthcare provider can compute the shared key S.

8 $$\:Ch=ROT\left(n\oplus\:{\text{t}}_{1},S\right)$$

(3) Next, the patient sends message M1 containing (Ch, A, $\:{\text{t}}_{1}$, n) through public channel. When the healthcare system receives this message, it checks the validity of the timestamp. If valid, it proceeds to calculate the shared key with the patient using the patient’s public key and the Chebyshev as shown in Eq. (9).

9 $$\:S={\text{T}}_{c}\left(A\right)\:\text{m}\text{o}\text{d}\:\text{p}$$

(4) Next, the healthcare system recovers the nonce from Ch, modifies it, and sends the updated nonce back to the patient, XORed with the shared key as described in Eq. (10).

10 $$\:M2=\text{n}{\prime\:}\oplus\:S$$

(5) Finally, when the patient receives the message, they use the shared key to recover the modified nonce and compare it with the original one to verify it.

After that, the patient begins the registration phase with the healthcare system, utilizing the shared key S for secure communication, allowing this phase to be completed over a public channel. The registration phase is carried out through the following steps:

1. The patient enters their personal information, including identity (ID), password (PW), and biometric data(B). The patient also selects a random value x, then computes WB and PB as described in Eqs. (11), (12).

11 $$\:WB=ROT(ID\oplus\:X,PW)$$

12 $$\:PB=ROT(ID\oplus\:X,{\text{h}}_{BIO}(B\left)\right)$$

Once these values are computed, the patient sends a registration request containing (ID, WB, PB) encrypted using the shared key S, to ensure secure transmission to the healthcare system.

2. Once the healthcare system receives the registration request, it decrypts the message using the shared key S. After decryption, the system computes Z as described in Eq. (13), and then selects random value r and compute Y using the Chebyshev chaotic map, as outlined in Eq. (14) for digital signature SN as in Eq. (15).

13 $$\:Z=ROT(ID\oplus\:WB\oplus\:PB,c)\:$$

14 $$\:\:\:\:\:Y={\text{T}}_{r}\left(w\right)\:\text{m}\text{o}\text{d}\:\text{p}\:\:$$

15 $$\:\:\:\:SN={(\:\text{r}\text{*}\text{c}}^{-1}+\text{Z}\:\:)\:\:\:\text{m}\text{o}\text{d}\:\:\text{p}\:$$

The healthcare system stores the patient’s ID and (WB⊕PB) in its database. Afterward, it sends a response to the patient that includes the unique identifier Z, the digital signature (Y, SN) and a timestamp.

3. Upon receiving the response, the patient verifies the digital signature (Y, SN) by computing Y′ and comparing it with the received Y to confirm the message’s authenticity, as described in Eq. (16).

16 $$\:Y{\prime\:}={\text{T}}_{SN-Z}\left(C\right)\:\text{m}\text{o}\text{d}\:\text{p}$$

If the verification is successful, the registration process continues. The patient then computes V as in (17) and stores it for future login. Additionally, the patient computes T to securely store Z.

17 $$\:V=ROT(PW\oplus\:X\:,PB)$$

**Fig. 2:**
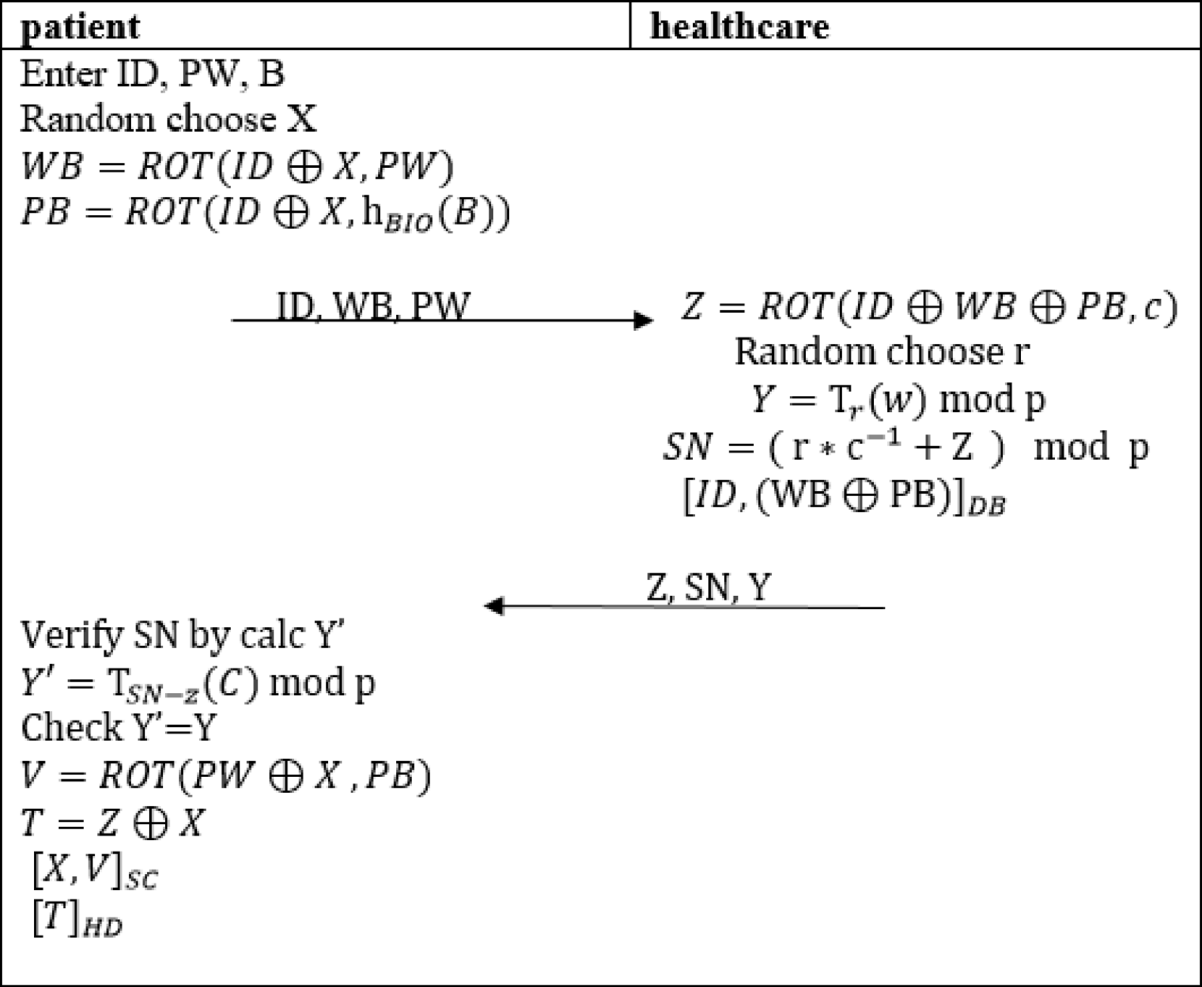
The registration phase of the proposed protocol.

Patient store (X, V) on smartcard and (T) on the intelligent healthcare device. The entire phase is illustrated in (Fig. 2).

#### Login and initial authentication

The patient begins the login process by inserting their smart card into the intelligent healthcare device and entering their credentials, including password (PW) and biometric data (B). The smart card then computes PB′ and subsequently calculates V′, comparing it with the stored V. If the values match, the authentication proceeds. Next, the patient selects a random value r and m, and computes F as described in Eq. (18). The patient subsequently recovers Z from T, generates the nonce, and then use it to calculates O as defined in Eq. (18a) and sends a message to the healthcare system containing (F, Z, O, t) encrypted with the shared key.

18 $$\:F=ROT(r\:,m)\:$$

18a $$\:O=I{D}^{{\prime\:}}\oplus\:ni\:\:$$

Upon receiving the message, the healthcare system first validates the timestamp. It then verifies the patient’s ID against its database by computing the ID from Z as described in Eq. (19) and comparing it with the received ID’, which is also obtained according to Eq. (18b).

18b $$\:I{D}^{{\prime\:}}=O\oplus\:ni\:$$

19 $$\:ID=ROT(z\:,c)\oplus\:\:(\text{W}\text{B}\oplus\:\text{P}\text{B})$$

The healthcare system then computes the shared key SK as defined in Eq. (20) and calculates the value A using Eq. (21), which will be used to ensure the integrity of the message. The patient can also compute the shared key SK on their side. The healthcare system sends a message to the patient containing (A//t) where A is concatenated with the timestamp t, encrypted with the shared key SK.

20 $$\:SK=F\oplus\:\:\text{Z}$$

21 $$\:A=ROT\left(F,t\right)$$

**Fig. 3:**
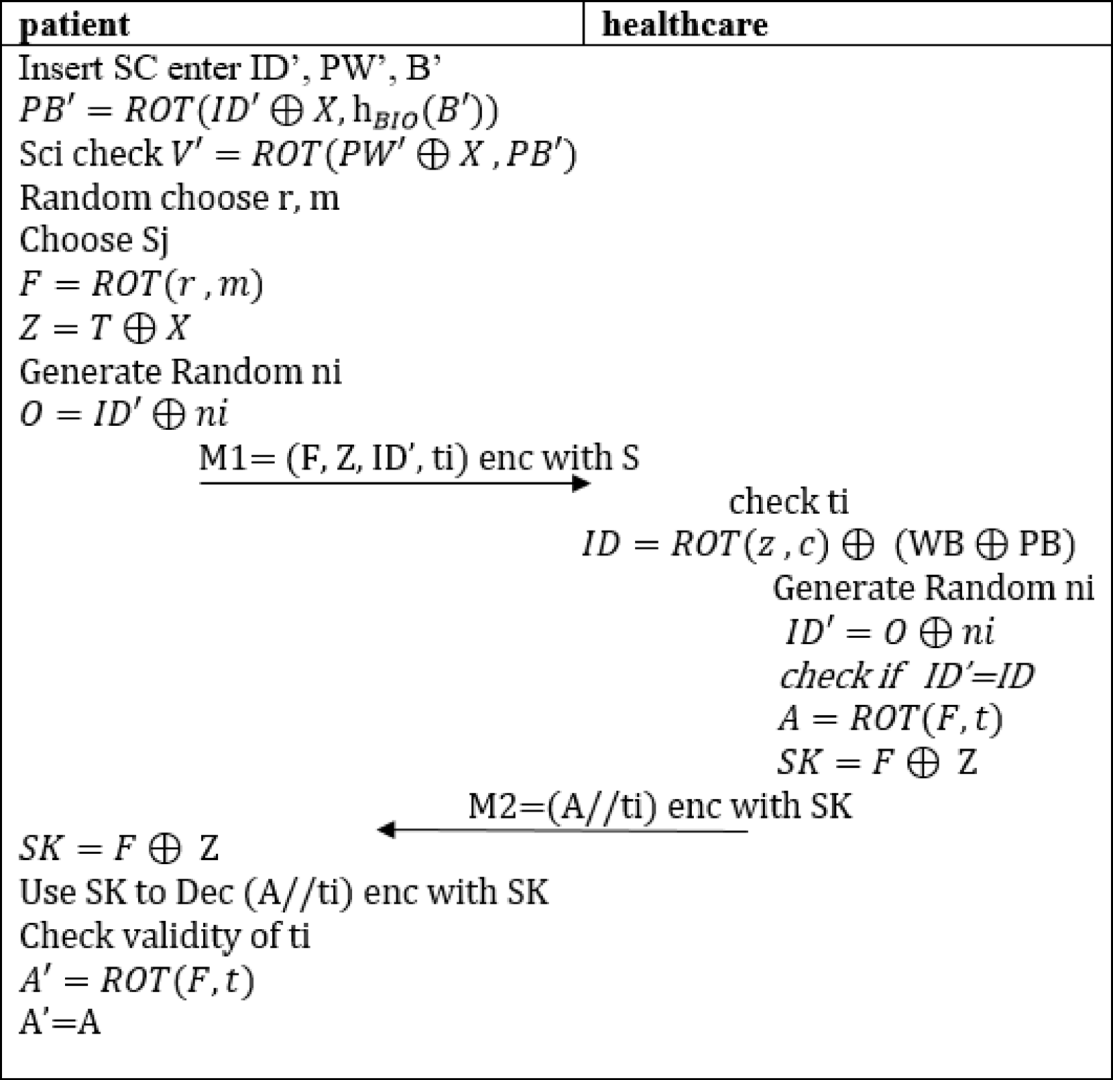
Login and initial authentication of the proposed protocol.

Once the patient receives a message, they begin to compute the shared key SK as defined in Eq. (22), along with the value A and compare it with received one to complete the authentication process if valid the shared key is established between patient and healthcare. The entire phase is illustrated in (Fig. 3).

22 $$\:SK=F\oplus\:\:\text{Z}$$

#### Rapid authentication

Using the previously established parameters r, m, F, and the session key SK, both the patient and the healthcare can quickly authenticate each other. These values allow for rapid mutual authentication and the computation of new shared session keys for secure communication in future sessions.

The patient begins the login process by inserting their smart card into the intelligent healthcare device and entering their credentials, including password (PW) and biometric data (B). The smart card then computes PB′ and subsequently calculates V′, comparing it with the stored V. If the values match patient compute $\:{\text{F}}_{E}$ as in Eq. (23) this valid for E < m if not the initial authentication is recalled.

Next, the patient computes Q as defined in Eq. (24) and sends it to the healthcare system.

23 $$\:{\text{F}}_{E}=ROT\left(r\:,m-E\right)\:$$

24 $$\:Q={(\text{F}}_{E})enc\:\text{S}\text{K}\:$$

Once the healthcare system receives Q, it decrypts the message using the shared key SK and verifies whether F=$\:{\text{F}}_{E}$. If the values do not match, the healthcare system update F with $\:{\text{F}}_{E}$ generates a new shared session key $\:{SK}_{NEW}$, as described in Eq. (25). The system then computes $\:{\text{Q}}_{1}$ according to Eq. (26) and sends it to the patient.

25 $$\:{SK}_{NEW}={\text{F}}_{E}\oplus\:\:\text{S}\text{K}$$

26 $$\:{\text{Q}}_{1}={(\text{F}}_{E})enc\:{SK}_{NEW}$$

**Fig. 4:**
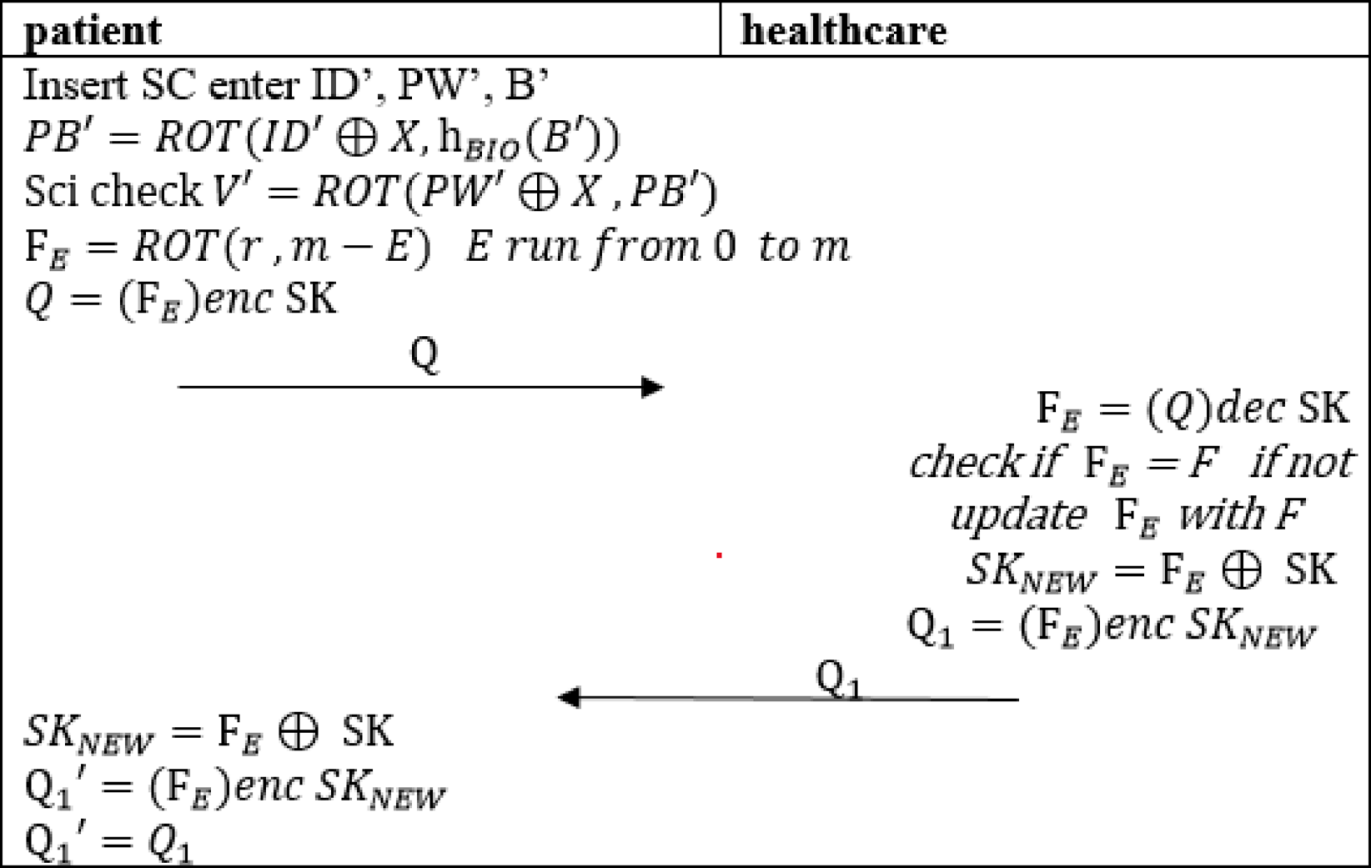
Rapid authentication of the proposed protocol.

Upon receiving $\:{\text{Q}}_{1}$, the patient calculates the new shared session key $\:{SK}_{NEW}$ using Eq. (27). Finally, the patient verifies $\:{\text{Q}}_{1}$ to successfully completing the authentication process. The entire phase is illustrated in (Fig. 4).

27 $$\:{SK}_{NEW}={\text{F}}_{E}\oplus\:\:\text{S}\text{K}$$

#### Password modification

Patients are allowed to update their password to improve security. Initially, the patient enters their credentials, including the old password (PW’) and biometric data (B’) then calculates V’ and verifies it. If the values match, the patient can request a new password ($\:{PW}_{NEW}$).

**Fig. 5:**
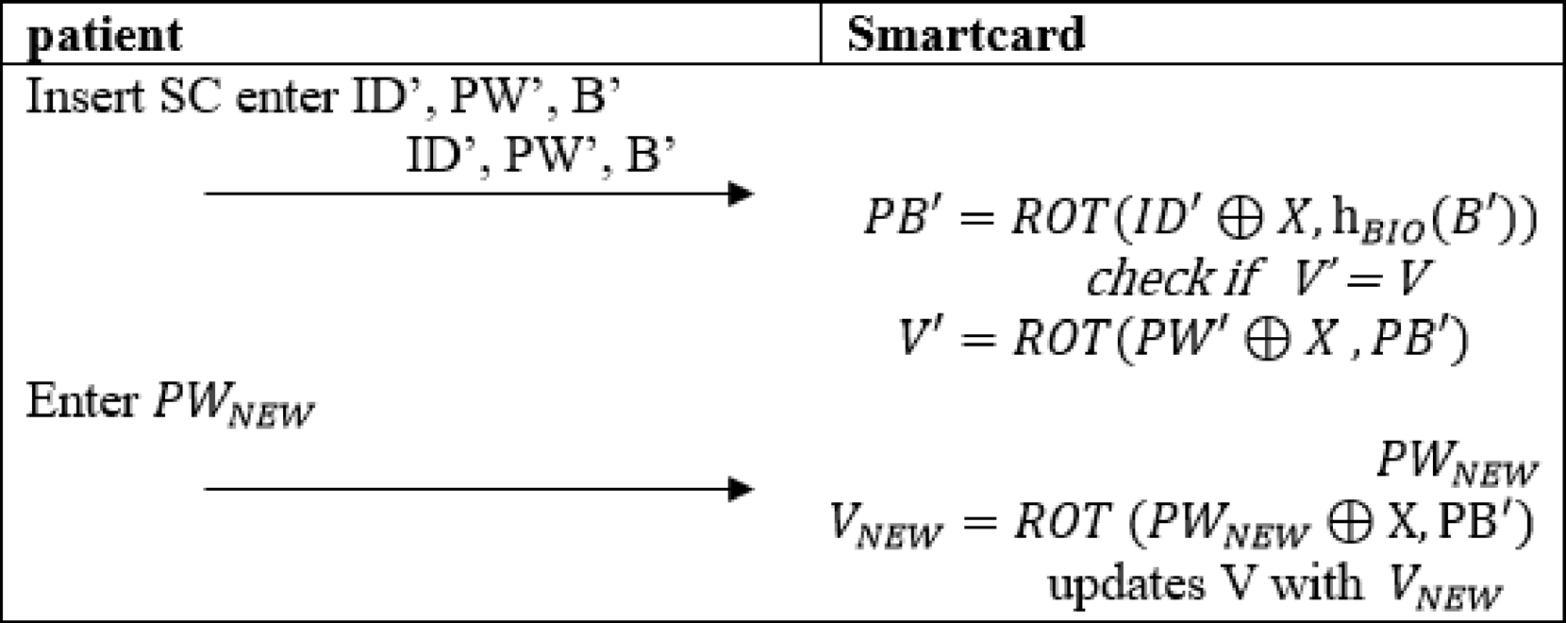
Password modification of the proposed protocol.

The system computes the new value ($\:{V}_{NEW}$) as in Eq. (28) next smart card updates V with $\:{V}_{NEW}$. The entire phase is illustrated in (Fig. 5).

28 $$\:{V}_{NEW}=ROT\:({PW}_{NEW}\oplus\:\text{X},\:{\text{P}\text{B}}^{{\prime\:}})$$

### Assumptions and operational conditions

#### System assumptions

The proposed protocol is designed for 6G-enabled healthcare environments, with the following assumptions:

- Network environment:
- Downlink speeds ≥ 1 Tbps with latency < 1 ms for critical healthcare applications.
- Support for massive IoT density (10⁷ devices/km²).
- Availability of PKI infrastructure for initial server authentication.
- Device capabilities:
- Patient devices (smart cards or intelligent healthcare IoT) are resource-constrained (≤ 100 MHz processors).
- Devices support lightweight operations such as XOR, bitwise rotation, and Chebyshev chaotic maps.
- Devices are equipped with secure biometric sensors with liveness detection.
- Healthcare servers possess quantum-resistant computational capabilities and maintain encrypted medical databases.

#### Security assumptions

- Trust model:
  - Patients and healthcare providers are initially mutually untrusted.
  - Smart cards are assumed tamper-resistant but can be stolen.
  - Biometric sensors are considered trustworthy, though readings may contain minor noise.
- Adversary capabilities:
  - The adversary can eavesdrop on all public communications.
  - The adversary may compromise either the password or the smart card, but not both simultaneously.
  - Biometric templates are assumed infeasible to extract.
  - The Chebyshev Chaotic Maps remain secure under the CDHP assumption.

#### Environmental constraints

- **Optimal conditions**:
  - End-to-end latency ≤ **5 ms**.
  - Packet loss rate < **0.1%**.
  - Continuous power availability for IoT and wearable devices.
- **Graceful degradation**:
  - Latency ≤ **50 ms**.
  - Temporary connectivity loss ≤ **10 s**.

#### Practical deployment requirements

- **Patient devices**:
  - Support for **IEEE 802.15.6 (WBAN)** or equivalent.
  - At least **128 KB secure storage**.
  - A **True Random Number Generator (TRNG)** for nonce generation.
- **Healthcare providers**:
  - Synchronized clocks (± 100 ms).
  - Enforcement of **secure key rotation policies**.

### Security properties achieved by the protocol

The security of the proposed protocol builds directly on the theoretical foundations described in Section III. The BioHashing mechanism ensures reliable user authentication by providing irreversibility, unlinkability, and tolerance to biometric variations (Table 4). The session key is established using Chebyshev chaotic maps, where its secrecy is guaranteed by the computational hardness of the Chebyshev Diffie–Hellman Problem (CDHP). The inclusion of random nonces in each protocol run ensures resistance against replay attempts and contributes to forward secrecy. Moreover, the integration of digital signatures ensures the authenticity and integrity of exchanged messages while resisting forgery and repudiation. Collectively, these mechanisms provide a strong security framework for the proposed protocol in 6G-enabled healthcare environments.

**Table 4:** Mapping of cryptographic foundations to achieve security goals.

Technique	Purpose in protocol	Security property achieved
BioHashing	Protects biometric template during authentication	Ensures irreversibility, guarantees unlinkability, supports fuzzy matching
Chebyshev chaotic maps	Session key generation and key exchange	Guarantees session key secrecy and provides forward secrecy
Random nonces	Incorporated in each protocol execution	Ensure resistance to replay attacks
Hash functions (SHA-512)	Validation of integrity and protocol computations	Provides data integrity and resistance to guessing attacks
Digital signatures	Verification of critical protocol messages	Guarantees authenticity, ensures integrity, and provides non-repudiation

## Security analysis

In this section, we will provide a detailed explanation of the various security features implemented by the proposed protocol. These features are designed to enhance the overall security of communications and data exchanges within the healthcare system.

### Secure mutual authentication

Upon reception of the login request message (F, Z, ID’, t) enc with S) from Pi, Sj decrypts the message, recovers the ID from Z, and compares it with ID’. If the confirmation is successful, Sj calculates the acknowledgment value A and the session key, then sends a message to the patient ((A//t) enc with SK). Pi also verifies the acknowledgment value A from Sj before confirming the shared session key. If either the ID or A verification fails, the communication session is terminated without generating a shared key, ensuring a Secure mutual authentication process in the proposed protocol.

### Ensuring perfect forward secrecy

Suppose an attacker unexpectedly. gains access to the session key from the current session and attempts to use it to compromise past communications between P_i_ and Sⱼ, the attempt would be unsuccessful. In each communication session, the session key SK is a unique nonce, derived from a random number r and processed through multiple operations such as rotation and XOR. This design ensures that the session key is unique and does not remain static. Consequently, the attacker cannot use the compromised key to target previous sessions. Therefore, the integrity and security of the communication remain intact, and this confirms the robustness of the proposed protocol.

### Ensuring user anonymity, untraceability, and message unlinkability

The identity ID_i_ of P_i_ is securely embedded within the encrypted request. Neither of the messages exchanged between Pi and Sj publicly reveals IDi, ensuring its privacy throughout the communication.

As a result, IDi remains concealed from any potential attacker during the communication process. It is a secret shared exclusively between Pi and Sj, Both M1 and M2 are nonce values, guaranteeing uniqueness in every communication session. As a result, an attacker cannot link any two transcripts exchanged by the same patient Pi, maintaining the privacy and security of each session.

### Protection against password guessing attacks

In this scenario, the attacker attempts to log in by entering a predicted password combined with an identity and biometric data. However, SCi verifies the value of V and promptly rejects the attacker’s candidate password, ensuring the system’s security. Suppose the attacker obtains the value Wb and attempts to guess Pi’s password using this value. However, Wb not only includes Pbi but also incorporates the identity IDi and a random number x. Without knowledge of IDi and x, the attacker cannot compute a valid Wb′ for comparison with Wb, making it computationally infeasible to guess the valid password. Our study.

is resilient to both online and offline password-guessing attacks. Additionally, the proposed protocol includes a password update function, further enhancing the security of PWi.

### Protection against impersonation attacks

Suppose an attacker unexpectedly obtains IDi and attempts to compute a login request to impersonate Pi. Due to the protocol’s inherent Protection Against password-guessing attacks, PWi remains secure and unrevealed to the attacker. Furthermore, Bi is exclusively known to Pi, ensuring additional protection. Even if the attacker possesses IDi, they cannot compute Wb and PB without access to PWi and Bi. Additionally, the attacker lacks the correct Z and Shared key needed to forge the ciphertext M1. Consequently, the proposed protocol effectively resists impersonation attacks, maintaining secure authentication.

### Protection against MITM attacks

During the login process, an attacker cannot encrypt a candidate message or forge M1 without knowledge of the shared key S. This ensures that the attacker cannot act as a middleman to alter the messages exchanged between Pi and Sj without being detected. Additionally, our protocol employs digital signatures based on the Chebyshev chaotic map to guarantee data integrity and mitigates forgery attacks. Any attempt by an attacker to modify the transmitted data is easily identified, as the attacker cannot alter or replicate the valid digital signature.

### Protection against replay attacks

An attacker might try to intercept and resend M1 to Sj in an attempt to execute a replay attack in future communication sessions. However, the protocol incorporates the use of a timestamp Ti, which verifies whether M1 has been reused. In other words, Ti ensures that M1 is valid for only a single login attempt. Similarly, Timestamp Tj is utilized to validate the authenticity of the message M2 sent to Pi. Additionally, the acknowledgment values A further verifies the legitimacy of both Pi and Sj. These mechanisms collectively ensure robust resistance to replay attacks, establishing the protocol’s security against such threats.

### Protection against desynchronization attacks

Within the proposed protocol, the acknowledgments A are computed using a random value and the timestamp Tj, which are deleted immediately after the communication session concludes. Neither Pi nor Sj retains any redundant parameters once the session ends. As a result, the protocol effectively resists desynchronization attacks, ensuring consistent and secure communication across sessions.

### Protection against stolen smart card attacks

IF an attacker steals SCi from Pi and performs a power analysis attack to retrieve all stored values within SCi. In the proposed protocol, neither the password PWi nor the biometrics Bi are directly stored in SCi. Even if the attacker obtains both HDi and SCi simultaneously and successfully bypasses the verification of SCi, they are still unable to compute a valid M1 without these critical parameters. Therefore, the proposed protocol effectively mitigates stolen smart card attacks.

### Protection against DoS attacks

During each login phase of the proposed protocol. SCi verifies the legitimacy of Pi by checking the input values. Specifically, SCi verifies **V** and instantly terminates the session if the validation fails. This ensures that an attacker cannot Overload the system with subsequent computational steps. Additionally, Sj verifies the freshness of the timestamp Ti immediately after decrypting M1. Repeated retransmissions of M1 to disrupt Sj’s services would be ineffective for the attacker, especially when the server side has redundant resources. To minimize the risk of DoS attacks and reduce communication costs, Ti is embedded in M1. Therefore, the conclusion is established.

## Comprehensive security validation

To ensure the robustness of the proposed authentication scheme, we conduct a two-layered formal security validation. First, we use automated symbolic analysis through the Scyther tool to verify protocol correctness under the Dolev–Yao adversary model. Then, we provide a mathematical security model to analyze the scheme under the computational model and standard cryptographic assumptions.

### Formal security evaluation using scyther

In this section, the security robustness of the proposed protocol is analyzed using a cryptographic verification tool. The primary objective of this tool is to evaluate the protocol’s security properties and identify any potential vulnerabilities that could be exploited by an attacker. For this purpose, **Scyther** is selected due to its free accessibility, high performance, and wide acceptance in academic research^22,24^. Scyther is a reliable simulation tool that provides a robust environment for analyzing security claims and uncovering vulnerabilities in cryptographic protocols.

#### Overview of scyther evaluation

The proposed protocol is modeled using the security protocol description language (SPDL), which is specifically designed for describing and analyzing cryptographic protocols. The simulation involves creating a formal representation of the protocol, including roles, functions, parameters, and security claims. The tool then analyzes the interactions between entities and evaluates the protocol’s resilience against various attack scenarios.

#### Key components in scyther simulation

1. **Role**:

Each entity involved in the protocol is defined as a “role.” A role can represent an initiator (e.g., Pi), a responder (e.g., Sj), or an attacker. Actions such as sending and receiving messages are specified within these roles.

2. **Functions**:

Cryptographic operations, such as hashing, encryption, digital signatures, and XOR functions, are explicitly defined. These functions simulate the behavior of the protocol’s cryptographic mechanisms.

3. **Parameters**:

Essential parameters, such as random nonces, timestamps, session keys, and identifiers (IDi), are specified. These parameters are crucial for modeling the protocol accurately.

4. **Security claims**:

Security properties to be verified by Scyther include:

- Authentication: Ensures mutual verification between Pi and Sj.
- Confidentiality: Protects sensitive data, such as session keys and user credentials.
- Freshness: Verifies the uniqueness of timestamps and nonces to resist replay attacks.
- Integrity: Detects any tampering of transmitted messages.

#### Simulation results

**Fig. 6:**
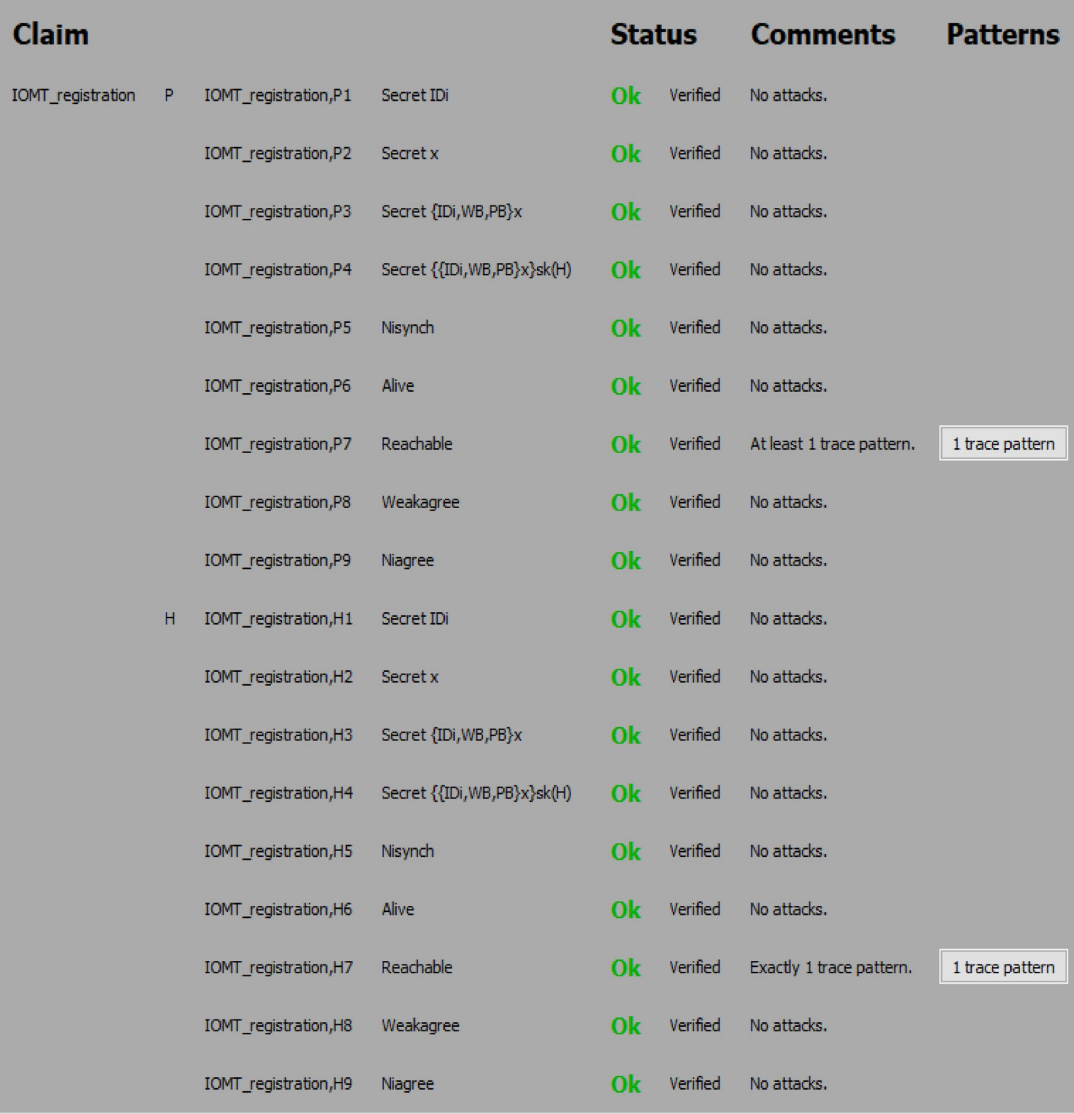
Security analysis result of the proposed protocol using Scyther.

**Fig. 7:**
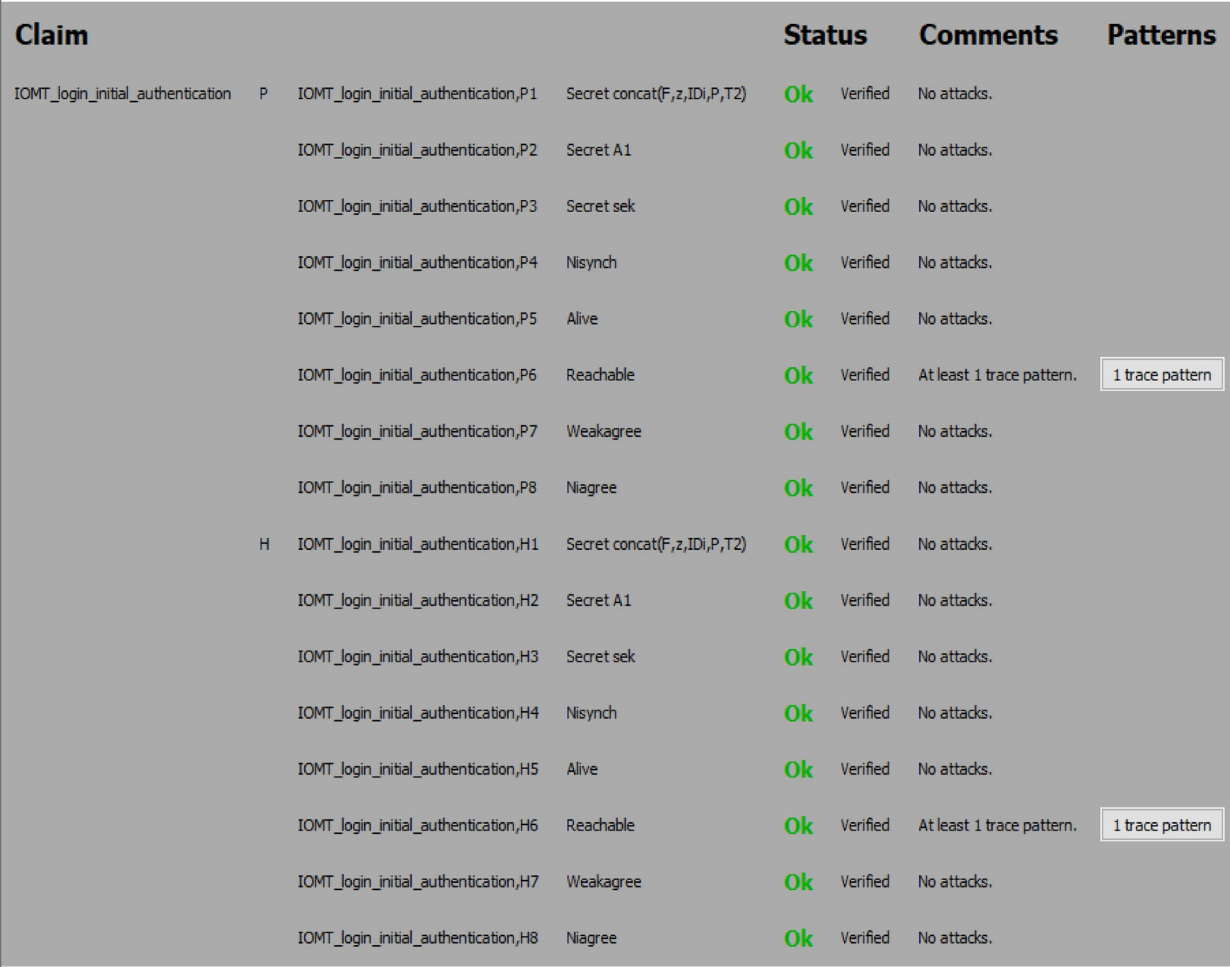
Security analysis result of the proposed protocol using Scyther.

**Fig. 8:**
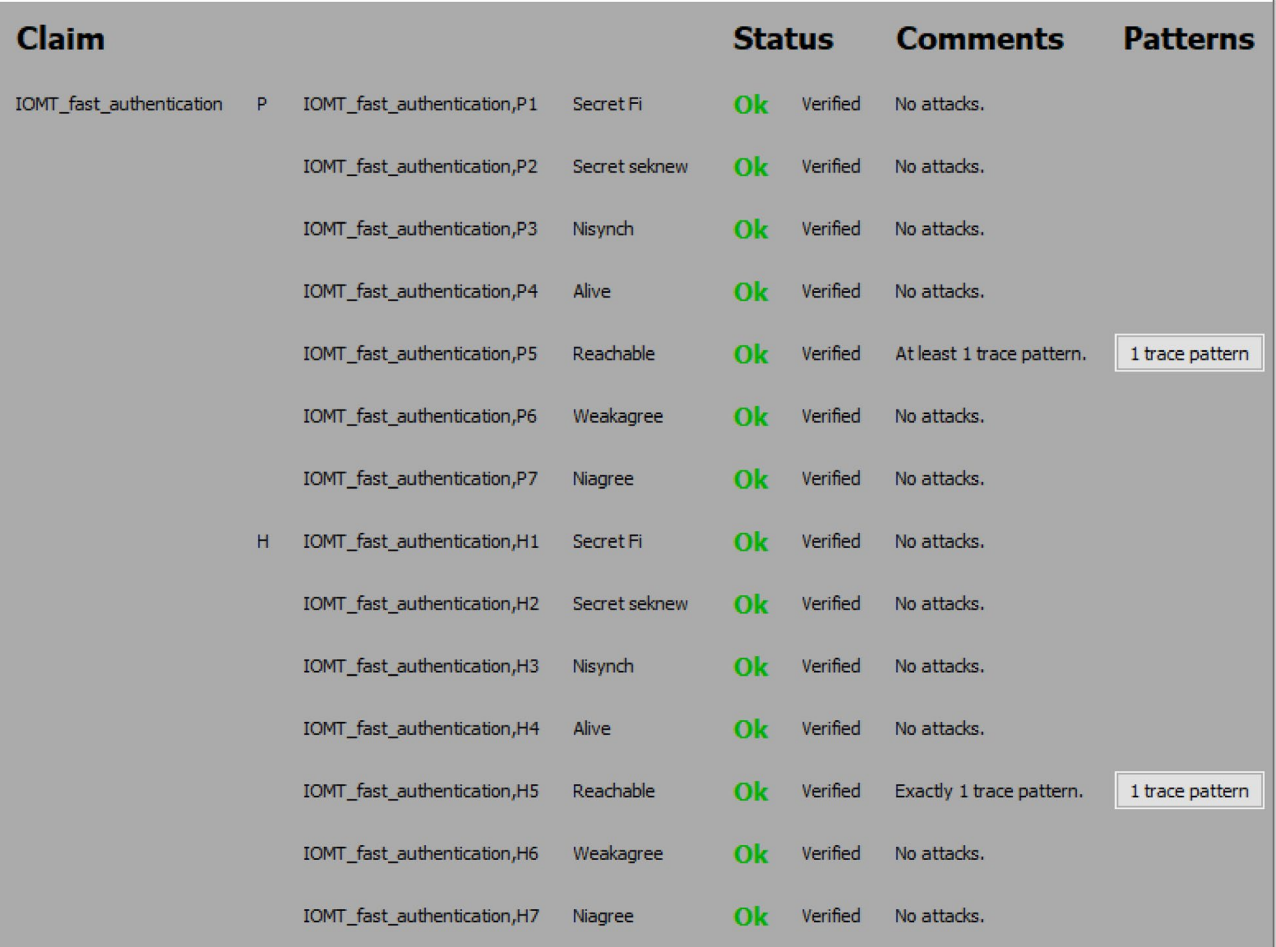
Security analysis result of the proposed protocol using Scyther.

The Scyther analysis focuses on evaluating the protocol against potential attack scenarios. Figures 6 and 7, and Fig. 8 summarize the claims and results:

#### Analysis of attack scenarios

Scyther simulated common attack scenarios, such as replay attacks, man-in-the-middle attacks, impersonation attacks, and eavesdropping. In all cases, the protocol demonstrated resilience and successfully thwarted the attacks.

#### Conclusion

(i) The Scyther-based formal verification confirms that the proposed protocol achieves its intended security objectives. By ensuring mutual authentication, confidentiality, integrity, and resistance to common attacks, the protocol has proved robust and secure for use in the 6G-enabled intelligent healthcare environment.

### Mathematical security model

To complement symbolic analysis, we establish a computational security model to mathematically prove the protocol’s resistance to well-known attacks. The adversary is modeled as a probabilistic polynomial-time (PPT) algorithm with access to all public messages.

#### Security assumptions

- Chebyshev Diffie–Hellman Problem (CDHP): Given ($\:{\text{T}}_{n}\left(x\right)\:$, $\:{\text{T}}_{m}\left(x\right)$), it is computationally infeasible to derive $\:{\text{T}}_{nm}\left(x\right)$ .
- Random Oracle Model (ROM): All hash functions are modeled as ideal random oracles, ensuring preimage and collision resistance.
- BioHash One-way Property: The BioHash transformation is assumed irreversible without knowledge of the transformation key.

#### Formal theorems

- Theorem 1 (mutual authentication): If an adversary A can impersonate a legitimate party with non-negligible probability ε, then A can solve the CDHP with probability ≥ ε. Since CDHP is intractable, impersonation success is negligible.
- Theorem 2 (session key secrecy): The probability that an adversary distinguishes the session key from a random value is bounded by: Pr[success] ≤ Pr[CDHP solved] + Pr[hash collision].

Both probabilities are negligible, ensuring session key indistinguishability.

- Theorem 3 (replay & MITM resistance): Nonces, timestamps, and session-specific Chebyshev parameters resist message reuse and manipulation. Thus, replay and man-in-the-middle attacks are infeasible under the ROM.
- Theorem 4 (forward secrecy): Even if long-term keys are compromised, past session keys remain secure due to the use of ephemeral Chebyshev values not derivable from previous transcripts.

#### Security summary

- Under the above assumptions, the proposed scheme achieves:
- ✔ Mutual authentication.
- ✔ Strong session key secrecy.
- ✔ Biometric privacy protection.
- ✔ Resistance to replay, MITM, and key-compromise impersonation attacks.
- ✔ Forward secrecy.

### Strong attack scenarios and threat model

To strengthen the practical relevance of our protocol, we consider strong attack scenarios, including cases where an adversary may steal a device, gain full control, access memory data, impersonate a legitimate user, or clone the device. The proposed scheme is designed to mitigate these threats as summarized in (Table 5).

**Table 5:** Attack scenarios vs. protocol defenses.

Attack scenario	Adversary capability	Protocol countermeasure
Device theft	Physical possession of patient device	Tamper-resistant storage; sensitive data encrypted; BioHash ensures biometric privacy even if device is stolen
Full device compromise	Full control over device operations	Separation of sensitive operations; ephemeral session keys; two-factor validation (password + BioHash)
Memory access	Access to RAM or local storage	Volatile memory for session keys; all sensitive data stored encrypted
User impersonation	Attempt to authenticate as legitimate user	Mutual authentication using nonces, timestamps, and Chebyshev maps; verification of session-specific digital signatures
Device cloning	Copy all device data to replicate device	Binding between device secrets and biometric identity; server-side detection of unauthorized devices

This threat model emphasizes that our protocol achieves strong security guarantees even under high-risk scenarios. The combination of BioHashing, ephemeral Chebyshev keys, digital signatures, and formal verification ensure robustness against these powerful adversaries.

## Performance analysis

In this section, we provide a thorough comparative analysis of the proposed protocol and several recently discussed protocols that share similarities with ours. The performance evaluation focuses on four key aspects: functionality, storage cost, communication cost, and computation cost.

### Functions

The comparison of various functionalities achieved by different protocols is summarized in (Table 6). The symbol ✓ indicates that a protocol supports a specific function, while × signifies that the function is not supported. It is evident that the proposed protocol offers more robust security properties and functionalities compared to the competing approaches. Notably, our protocol integrates fast authentication mechanisms within the proposed 6G-IoE intelligent healthcare environment.

**Table 6:** Comparison on functionality.

Functions	^25^	^13^	^1^	^9^	Our
Protection against password guessing attacks	✓	✓	✓	✓	✓
Protection against MITM attacks	✓	✓	✓	✓	✓
Protection against replay attacks	✓	✓	×	×	✓
Protection against desynchronization attacks	✓	✓	✓	✓	✓
Protection against inside attacks	×	✓	✓	✓	✓
Protection against stole card attacks	×	✓	×	✓	✓
Provide mutual authentication	✓	✓	✓	✓	✓
Provide perfect forward secrecy	✓	✓	✓	✓	✓
Provide patient anonymity	✓	✓	✓	✓	✓
Provide patient untraceability	×	✓	✓	✓	✓
Provide message unlinkability	×	✓	✓	✓	✓
Provide three factor authentication	×	✓	✓	✓	✓
Provide mathematical security proof	×	✓	✓	✓	✓
Provide security simulation with Scyther	×	×	×	×	✓
Provide rapid authentication	×	×	×	✓	✓
Provide password update function	×	×	×	✓	✓
Provide UCSSO solution	✓	×	✓	✓	✓
Provide nonrepudiation	×	×	×	×	✓
Using lightweight cryptographic tool	×	×	×	×	✓
Protection against DOS attacks	✓	✓	×	×	✓

### Comparison based on network structure

To securely transfer secret parameters during the registration process or establish communication within the healthcare system, existing schemes rely on secure channels, which significantly increase network deployment costs. However, our proposed protocol eliminates the need for a secure channel, making it more efficient and cost-effective. This comparison is illustrated in Table 7, highlighting the differences between our approach and existing schemes in terms of secure channels.

**Table 7:** Comparison on functionality.

Functions	^25^	^13^	^1^	^9^	Our
Secure channel used in registration	✓	✓	✓	✓	×

### Storage and communication cost

We define the key parameters used in the comparison to ensure strong security and efficiency. Chebyshev polynomials are set to 1024 bits, while symmetric encryption and decryption operate with a block size of 256 bits. Each identity, password, and biometric data entry has a length of 128 bits, whereas random numbers and hash values require 160 bits. Additionally, timestamps are assigned lengths of and 32 bits, respectively.

**Fig. 9:**
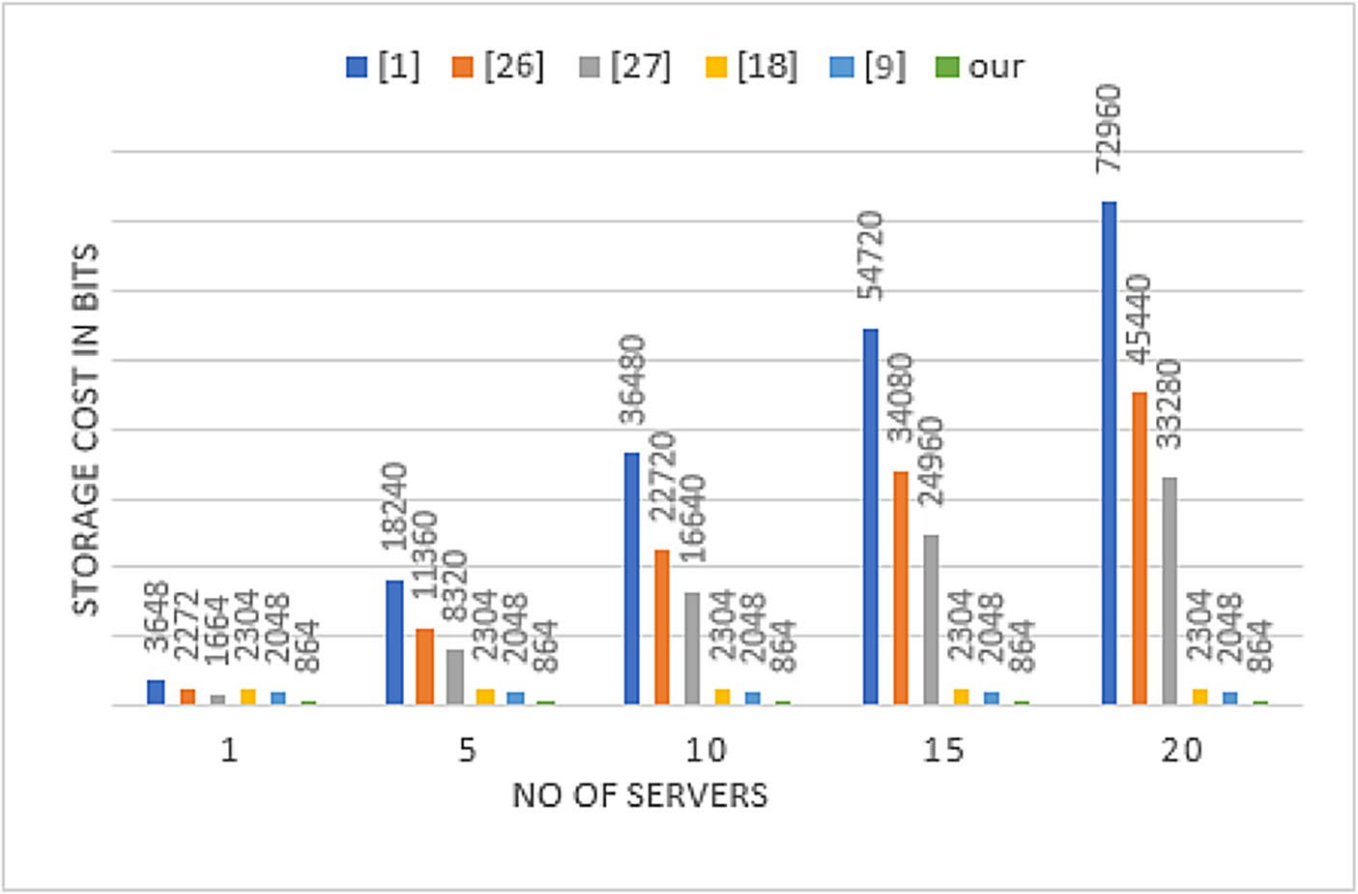
Comparison of storage cost.

The storage cost is determined based on the total size of the parameters stored by Pi and Sj after the registration phase. In our proposed protocol, Pi stores {T} on the device and {$\:X,V$} on the smart card, leading to a total storage requirement of 576 bits. Meanwhile, Sj retains the identity IDi and $\:(\text{W}\text{B}\oplus\:\text{P}\text{B})$ of each Pi in its database, requiring just 288 bits. Storage costs for other protocols^1,9,18,26,27^ are calculated similarly and summarized in Table 8, where it is evident that our protocol achieves the lowest storage overhead among the compared schemes. Furthermore, since our protocol incorporates a single sign-on (SSO) solution, the storage cost on the client side remains independent of the number of servers. Instead of requiring separate credentials for each server, a single set of credentials allows access to multiple servers, significantly enhancing storage efficiency and reducing overhead. Figure 9 provides a detailed comparison of the storage cost across different methods, illustrating how our proposed approach achieves lower storage overhead compared to existing schemes.

**In terms of communication cost**, during the initial authentication phase, when Pi enters IDi, PWi, Bi (retrieved from HDi) into SCi, it incurs a cost of 384 bits. After successful verification, SCi transmits and PBi back to (X, V) Pi for further computations, adding an additional 320-bit cost. This results in a total communication overhead of **704 bits**. Similarly, in the fast authentication phase, the communication cost is reduced, requiring only **384 bits** for the entire process. We also consider the total number of communication rounds, and the overall length of all transcripts exchanged between Pi and Sj during each authentication session. In our protocol, the transcripts include (M1; M2) in the initial authentication phase, which together consume 1280 bits, and (Q, Q1) in the fast authentication phase, amounting to 512 bits in total.

A detailed comparison of the communication cost across different protocols is presented in (Table 9). It is evident that the proposed protocol is among the most efficient in terms of initial authentication. Furthermore, during the rapid authentication phase, it incurs the lowest communication overhead among the compared schemes. Since most existing protocols rely on smart card mechanisms to ensure high security, the associated communication costs and round-trip times are considered reasonable within secure authentication frameworks.

**Table 8:** Comparison of storage cost of each entity.

Protocol	Pi side (bits)	Sj side (bits)
^1^	1824	1824
^26^	1664	608
^27^	1504	160
^18^	2176	128
^9^	1920	128
Our	576	288

**Table 9:** Comparison of communication cost.

Protocol	Cost btw Sci and Pi		Cost btw Sj and pi
^1^	544		1440
^26^	1760		3072
^27^	1472		1568
^18^	544		5736
^9^	Initial	704	1280
	Rapid	384	512
Our	Initial	704	1280
	Rapid	384	512

### Computation cost

In this section, we evaluate the computation cost of the proposed protocol by analyzing the cryptographic operations involved in authentication. To minimize both processing time and complexity, we utilize lightweight operations such as XOR and rotation instead of computationally intensive hash functions. Compared to existing schemes, our protocol significantly reduces computational overhead, particularly in the fast authentication phase, making it highly suitable for resource-constrained devices in a 6G-enabled intelligent healthcare environment.

### Experimental setup and device context

To ensure that the reported performance metrics reflect realistic conditions, the evaluation was carried out on a platform emulating a typical healthcare IoT device. The specifications include an ARM Cortex-M4 microcontroller operating at 100 MHz with 256 KB RAM, representative of modern intelligent healthcare devices and wearables. The operations were timed and analyzed using the Wolfram Mathematica environment, configured to mimic the computational capacity of such a device. This setup validates that the proposed protocol is not only theoretically efficient but also practically deployable in 6G-enabled healthcare systems.

A detailed breakdown of the computation cost is presented in Table 10, demonstrating the efficiency of our approach.

We assume that the time required for computing XOR and rotation operations is negligible due to their high-speed execution, as supported by^9,22^. According to^28^, the execution times of a BioHash function and a one-way hash function are comparable, and for simplicity, they are considered equivalent in this analysis.

To evaluate the computation cost, we define the following cryptographic functions and operations:

- $\:{\text{T}}_{CH}$: Time required to compute a Chebyshev chaotic polynomial mapping.
- $\:{\text{T}}_{F}\:\:$: Time needed to perform a fuzzy extractor operation.
- $\:{\text{T}}_{ECM}$: Time taken for elliptic curve point multiplication.
- $\:{\text{T}}_{ECA}$: Time required for elliptic curve point addition.
- $\:{\text{T}}_{SED}$: Time used for symmetric encryption or decryption.
- $\:{\text{T}}_{AESD}$: Time needed for asymmetric encryption or decryption.
- $\:{\text{T}}_{M}$: Time required for modular squaring computation.
- $\:{\text{T}}_{QR}$: Time taken to compute a square root modulo N.
- $\:{\text{T}}_{H}$: Time needed to compute a hash function.

The computation cost comparison for different protocols during a single communication session is presented in (Table 10). Assuming that all time bounds set by the servers are identical and E is fixed at 5. The results demonstrate that our protocol, particularly in the rapid authentication phase, achieves the highest efficiency, significantly reducing computational overhead while maintaining security guarantees.

**Table 10:** Computational cost comparison for a single session.

Protocol		Time complexity of Pi side	Estimated time of Pi side in ms	Time complexity of Sj side	Estimated time of Sj side in ms	Total estimated time ms
^1^		$\:{\text{T}}_{M}+{\text{T}}_{SED}+{9\text{T}}_{H}$	0.00744	$\:{\text{T}}_{QR}+{2\text{T}}_{SED}+{5\text{T}}_{H}$	1.17353	1.18097
^13^		$\:{9\text{T}}_{ECM}+40{\text{T}}_{H}+{\text{T}}_{F}$	5.1076	$\:{3\text{T}}_{ECM}+{8\text{T}}_{H}$	1.52952	6.63712
^18^		$\:{2\text{T}}_{CH}+2{\text{T}}_{SED}+{7\text{T}}_{H}$	0.06353	$\:{2\text{T}}_{CH}+2{\text{T}}_{SED}+{5\text{T}}_{H}$	0.06215	0.12568
^9^	Initial	$\:{\text{T}}_{M}+{\text{T}}_{SED}+{9\text{T}}_{H}$	0.00744	$\:{\text{T}}_{QR}+{2\text{T}}_{SED}+{8\text{T}}_{H}$	1.17560	1.18304
	Rapid	$\:2{\text{T}}_{SED}+{5\text{T}}_{H}$	0.00453	$\:2{\text{T}}_{SED}+{2\text{T}}_{H}$	0.00246	0.00699
Our	Initial	$\:{4\text{T}}_{X}+{4\text{T}}_{R}+2{\text{T}}_{SED}+{2\text{T}}_{H}$	0.00246	$\:{2\text{T}}_{X}+{2\text{T}}_{R}+{2\text{T}}_{SED}+{\text{T}}_{H}$	0.00177	0.00423
	Rapid	$\:{3\text{T}}_{X}+{3\text{T}}_{R}+2{\text{T}}_{SED}+{\text{T}}_{H}$	0.00177	$\:{\text{T}}_{X}+2{\text{T}}_{SED}$	0.00108	0.00285

According to^1,9^ $$\:{\text{T}}_{CH}=.02881ms$$, $$\:{\text{T}}_{F}$$=.508ms, $$\:{\text{T}}_{ECM}=.508ms$$, $$\:{\text{T}}_{ECA}=.0069ms$$, $$\:{\text{T}}_{SED}=.00054ms$$, $$\:{\text{T}}_{ASED}=$$1.169ms, $$\:{\text{T}}_{M}=.00069ms$$, $$\:{\text{T}}_{QR}=$$1.169ms, $$\:{\text{T}}_{H}=.00069ms$$

### Performance–security trade-off

A key design goal of the proposed protocol is to achieve strong security guarantees without imposing high computational costs on resource-constrained healthcare devices. To this end, we combined Chebyshev chaotic maps and BioHashing, both of which are lightweight in nature.

- Chebyshev maps rely on iterative polynomial evaluations modulo *p*, which require only basic arithmetic operations. Compared to elliptic curve cryptography, they offer reduced computational complexity while still relying on a hard mathematical problem (CDHP).
- BioHashing involves a single hash computation and XOR operation, which is significantly less expensive than fuzzy extractor mechanisms that require error-correction coding.

Our evaluation (see Table 10) shows that the proposed scheme achieves an average authentication time of **0.00354 ms** and reduces storage requirements by **57%** compared with existing three-factor protocols. These results demonstrate that the protocol maintains a strong level of security while remaining efficient and practical for devices with limited computational capacity (≤ 100 MHz processors).

## Conclusion

This paper presented a lightweight and secure three-factor authentication protocol for 6G-enabled healthcare systems. By combining smart cards, passwords, and biometric verification through BioHashing, the scheme provides **robust** identity assurance while remaining efficient for resource-constrained intelligent healthcare devices. The use of Chebyshev chaotic maps and lightweight cryptographic operations **balances** efficiency and security in these constrained environments.

In addition to these advantages, the proposed protocol introduces a fast authentication mechanism through parameter reuse and eliminates the requirement of a pre-established secure channel during registration, thereby enhancing practicality in real-world deployment. Furthermore, it provides a novel certificate-less, user-centric framework for multi-server healthcare environments. These contributions collectively establish the originality of the work and reinforce its suitability for latency-sensitive healthcare applications in 6G networks.

### Security evaluations were conducted using two complementary approaches

Formal verification using the Scyther tool confirmed the protocol’s resilience against major attacks, including replay, impersonation, and man-in-the-middle threats, validating critical properties such as secrecy and freshness. Additionally, the mathematical model provided analytical evidence of resistance against key-compromise attacks.

Overall, the protocol offers a well-balanced solution that combines security, efficiency, and scalability, making it highly suitable for next-generation healthcare infrastructures.

Future research will focus on real-world deployment across heterogeneous healthcare IoT environments, extending the model to support multi-modal biometrics, and integrating post-quantum cryptographic primitives to ensure long-term resilience against quantum-capable adversaries.

## Data Availability

The datasets generated and analyzed during the current study are available from the corresponding author on reasonable request.
